# Supplementary figures and images for: Methylglyoxal mutagenizes single-stranded DNA via Rev1-associated slippage and mispairing
Source: bioRxiv. 2025 Mar 18:2025.03.18.643935. Preprint. [Version 1] doi: 10.1101/2025.03.18.643935 (PMC11956917; doi:10.1101/2025.03.18.643935)

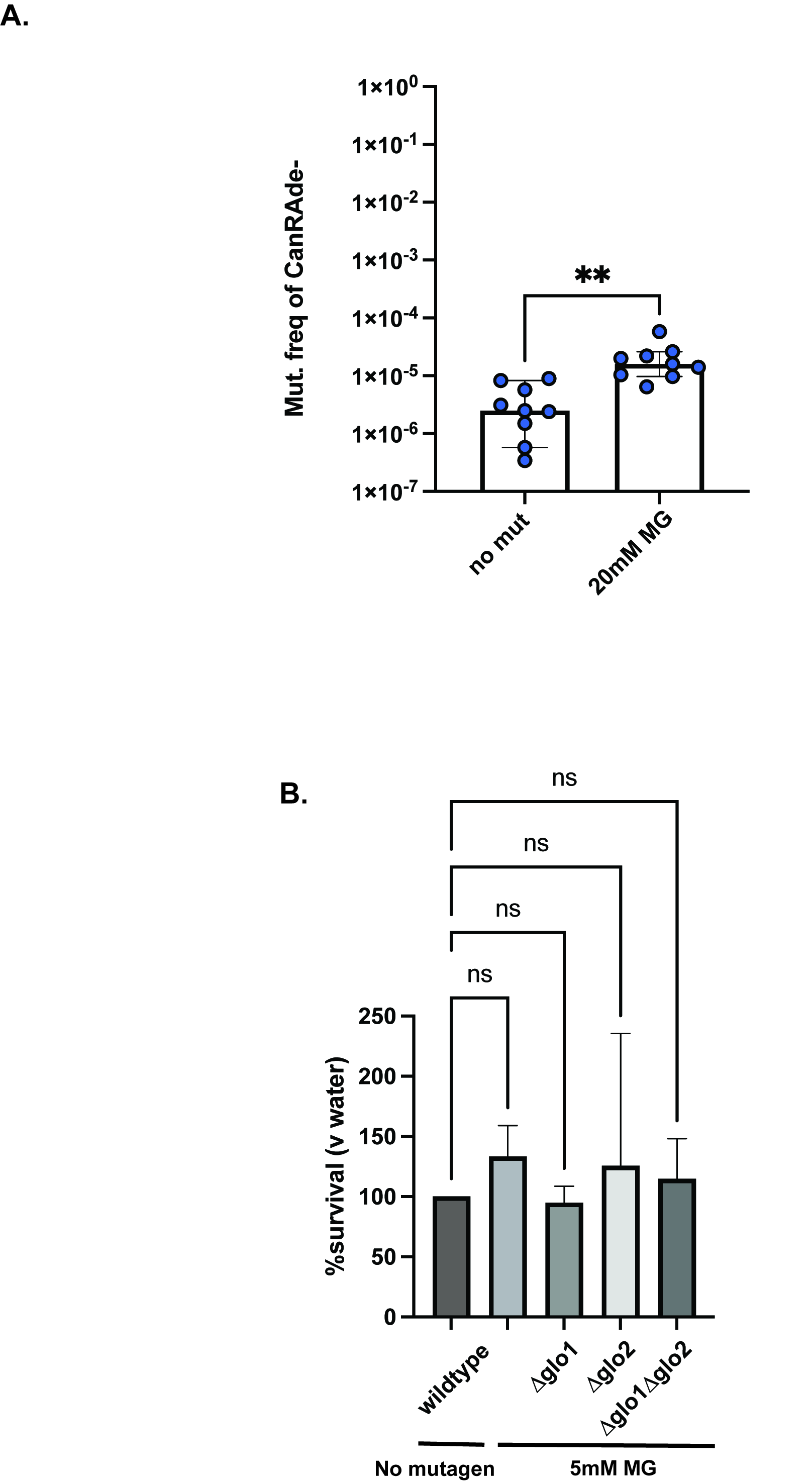

Supplement: Supplement 1 [file media-1.tif]

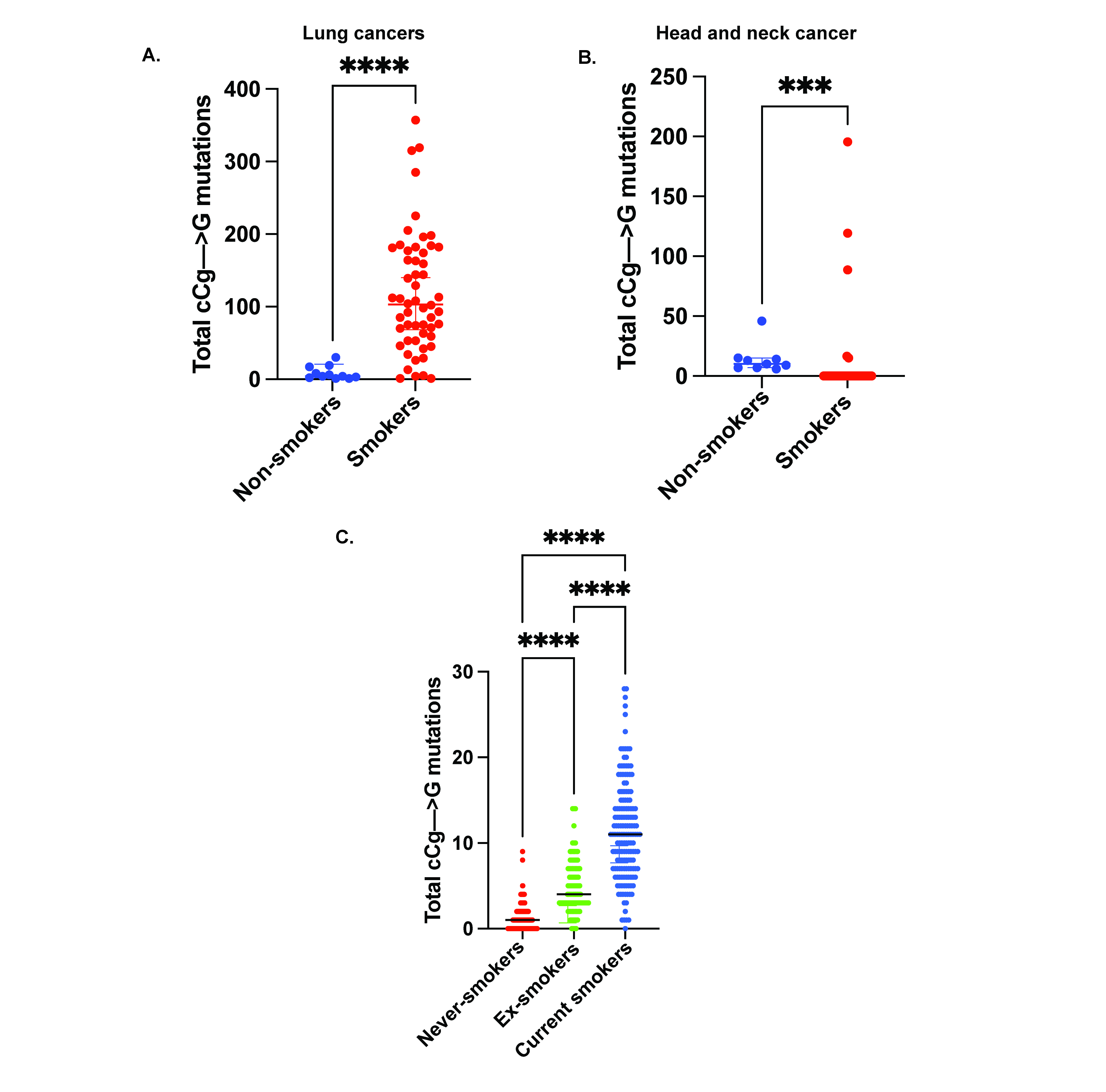

Supplement: Supplement 2 [file media-2.tif]

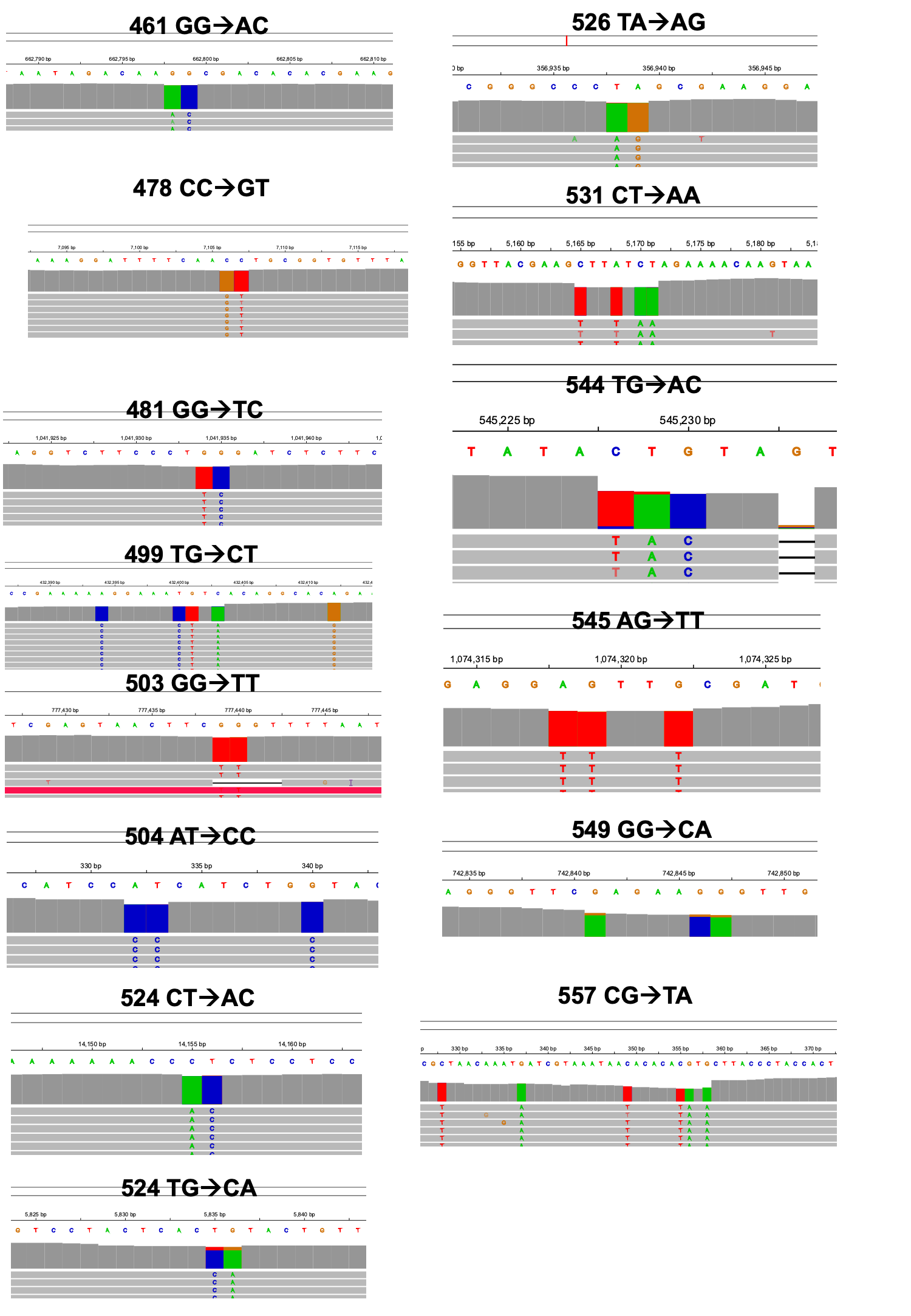

Supplement: Supplement 13 [file media-13.tif]

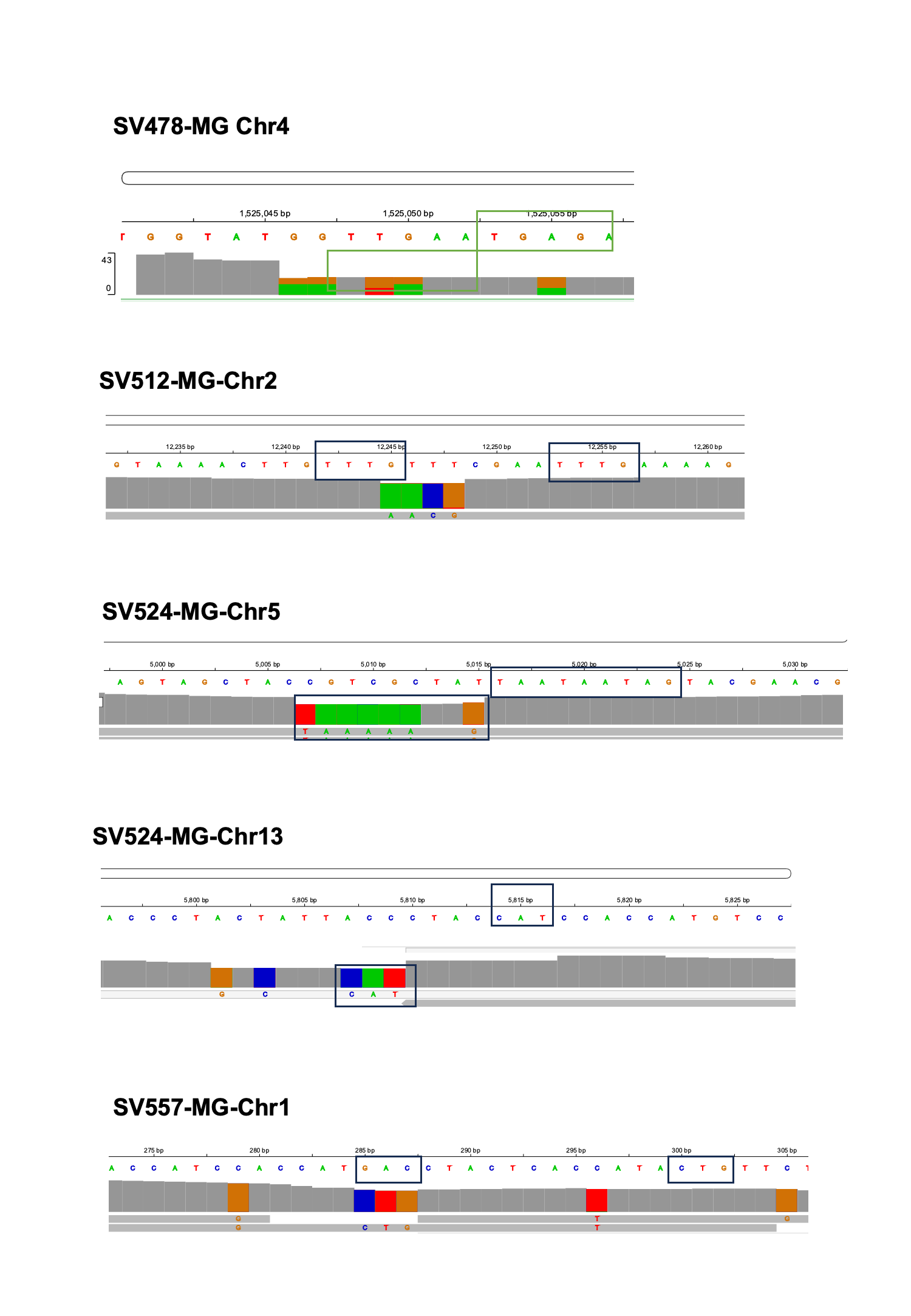

Supplement: Supplement 14 [file media-14.tif]
