# Supplementary material for: Methylglyoxal mutagenizes single-stranded DNA via Rev1-associated slippage and mispairing
Source: bioRxiv. 2025 Mar 18:2025.03.18.643935. Preprint. [Version 1] doi: 10.1101/2025.03.18.643935 (PMC11956917; doi:10.1101/2025.03.18.643935)
Supplement: 1 [file NIHPP2025.03.18.643935V1-supplement-1.pdf]

bioRxiv preprint doi: https://doi.org/10.1101/2025.03.18.643935; this version posted March 18, 2025. The copyright holder has placed this preprint (which was not certified by peer review) in the Public Domain. It is no longer restricted by copyright. Anyone can legally share, reuse, remix, or adapt this material for any purpose without crediting the original authors.

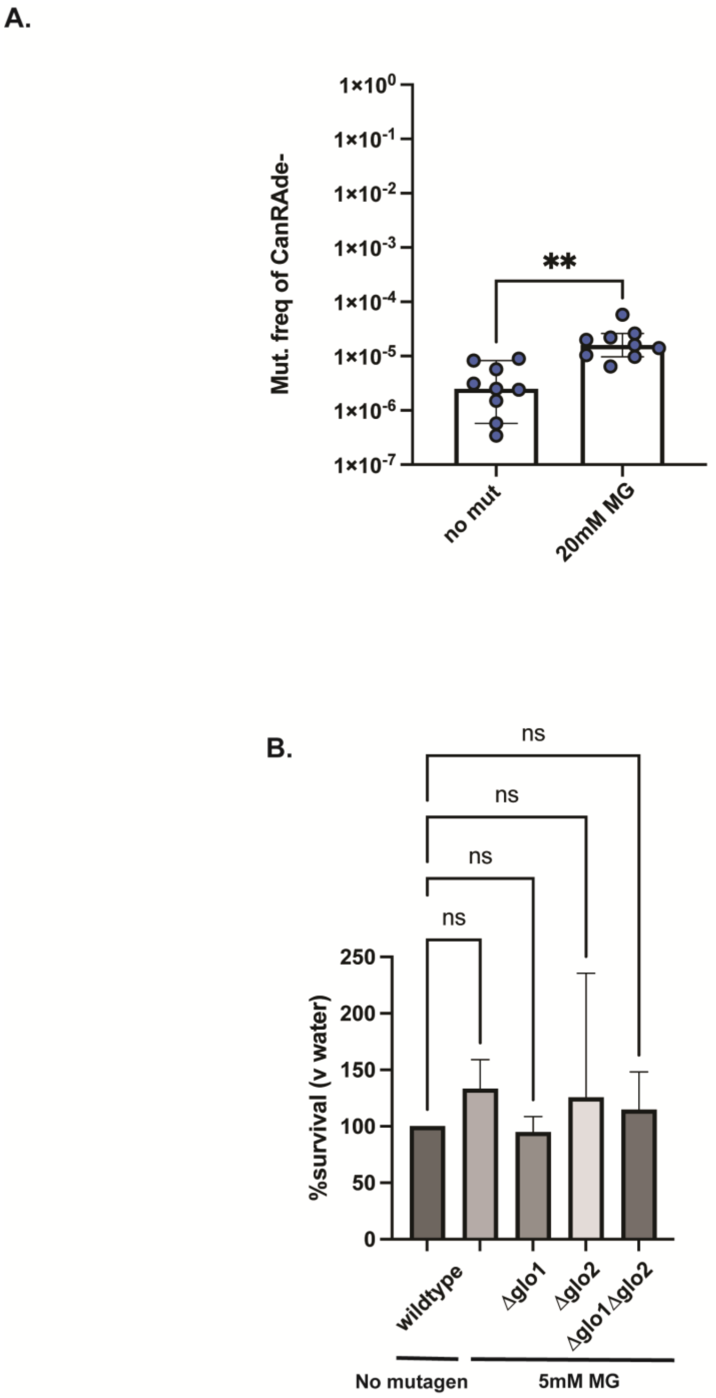

<u>Figure S1.</u> A.Can^{R}Ade$^-$ mutation frequencies of wildtype strains in response to 1hr, 20mM MG treatment. ** represents a statistically significant difference in median frequencies, indicating a p-value <=0.005 based on an unpaired two-tailed Student's t-test. B. Viability of strains with MG treatment. Ns-non-significant based on an ordinary one-way ANOVA

bioRxiv preprint doi: https://doi.org/10.1101/2025.03.18.643935; this version posted March 18, 2025. The copyright holder has placed this preprint (which was not certified by peer review) in the Public Domain. It is no longer restricted by copyright. Anyone can legally share, reuse, remix, or adapt this material for any purpose without crediting the original authors.

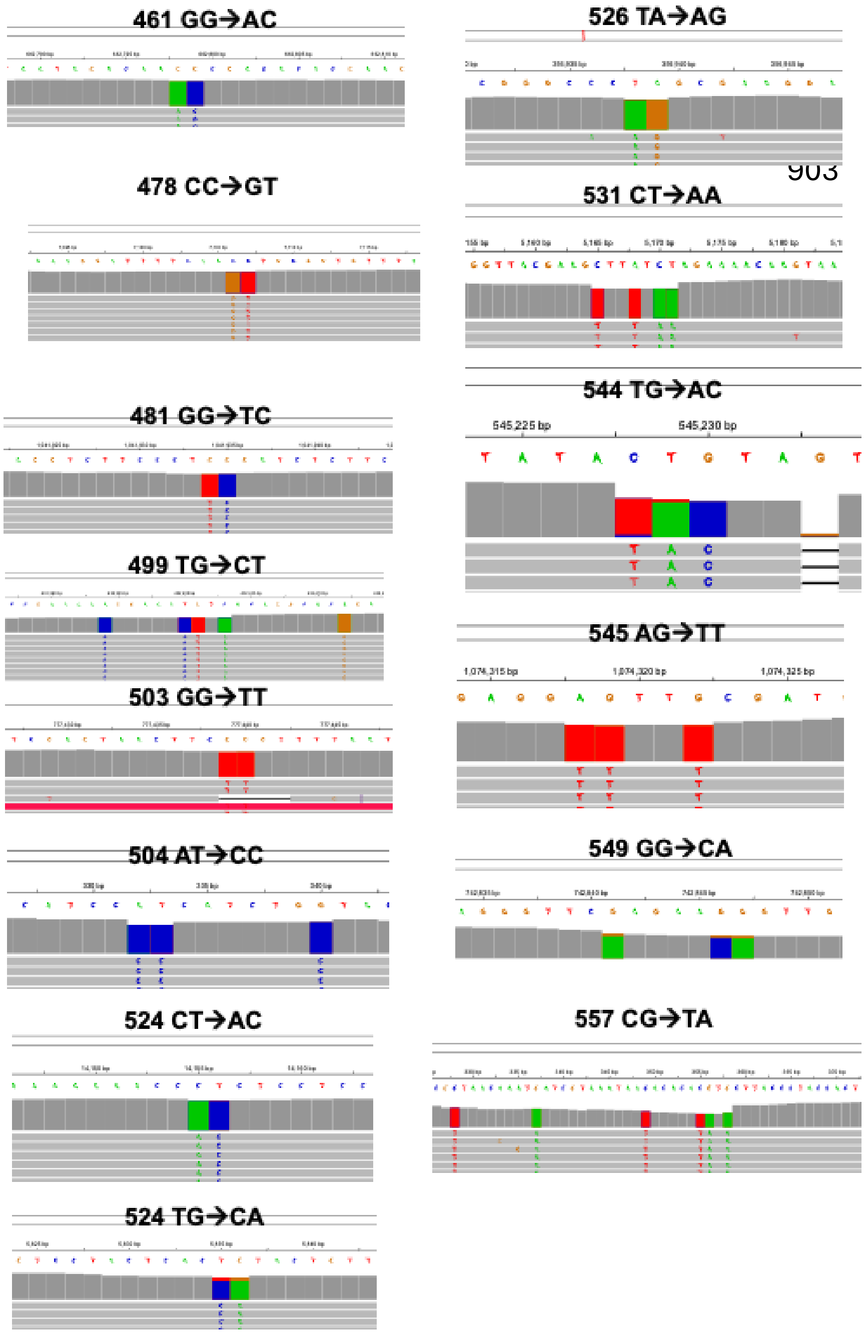

Figure S2. MG-associated double-base substitutions showing putative template realignment with bases downstream from the reference base and the resulting DBS denoted over each plot. Chromosome plots were generated using the Integrative Genome Viewer (https://igv.org/app/).

bioRxiv preprint doi: https://doi.org/10.1101/2025.03.18.643935; this version posted March 18, 2025. The copyright holder has placed this preprint (which was not certified by peer review) in the Public Domain. It is no longer restricted by copyright. Anyone can legally share, reuse, remix, or adapt this material for any purpose without crediting the original authors.

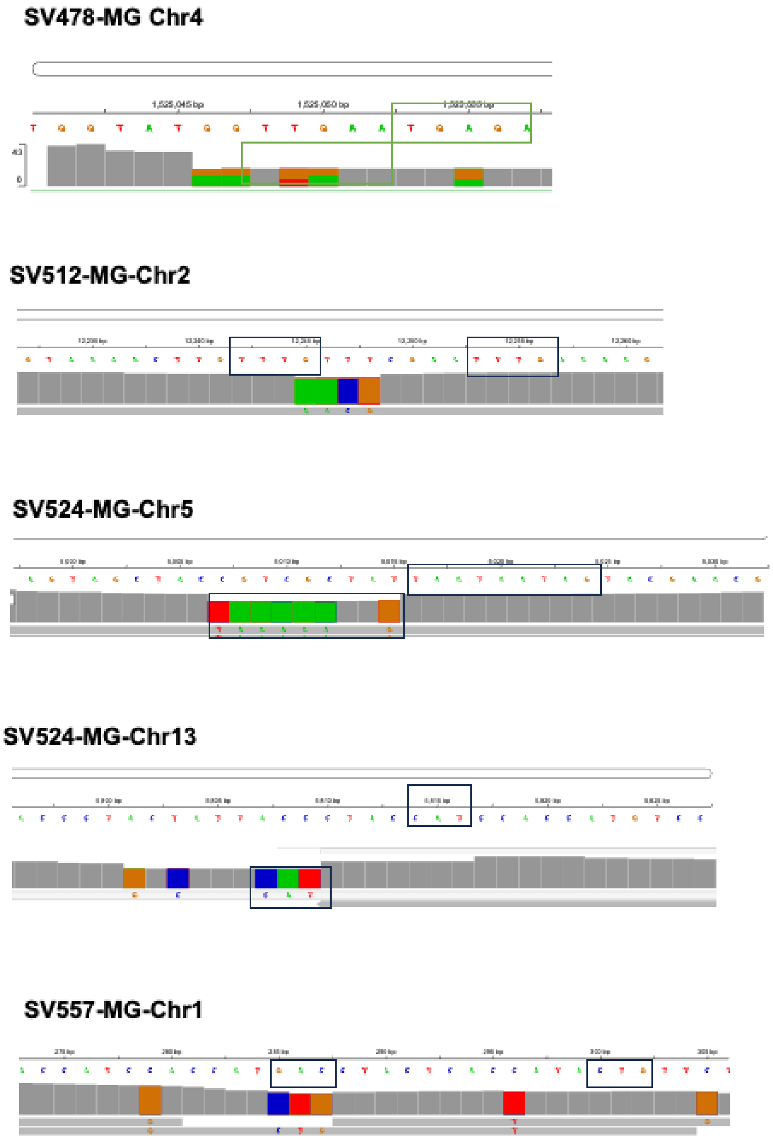

Figure S3. MG-associated multi-base substitutions showing putative template realignment with bases downstream from the reference base and copying. Chromosome plots were generated using the Integrative Genome Viewer (https://igv.org/app/).

bioRxiv preprint doi: https://doi.org/10.1101/2025.03.18.643935; this version posted March 18, 2025. The copyright holder has placed this preprint (which was not certified by peer review) in the Public Domain. It is no longer restricted by copyright. Anyone can legally share, reuse, remix, or adapt this material for any purpose without crediting the original authors.

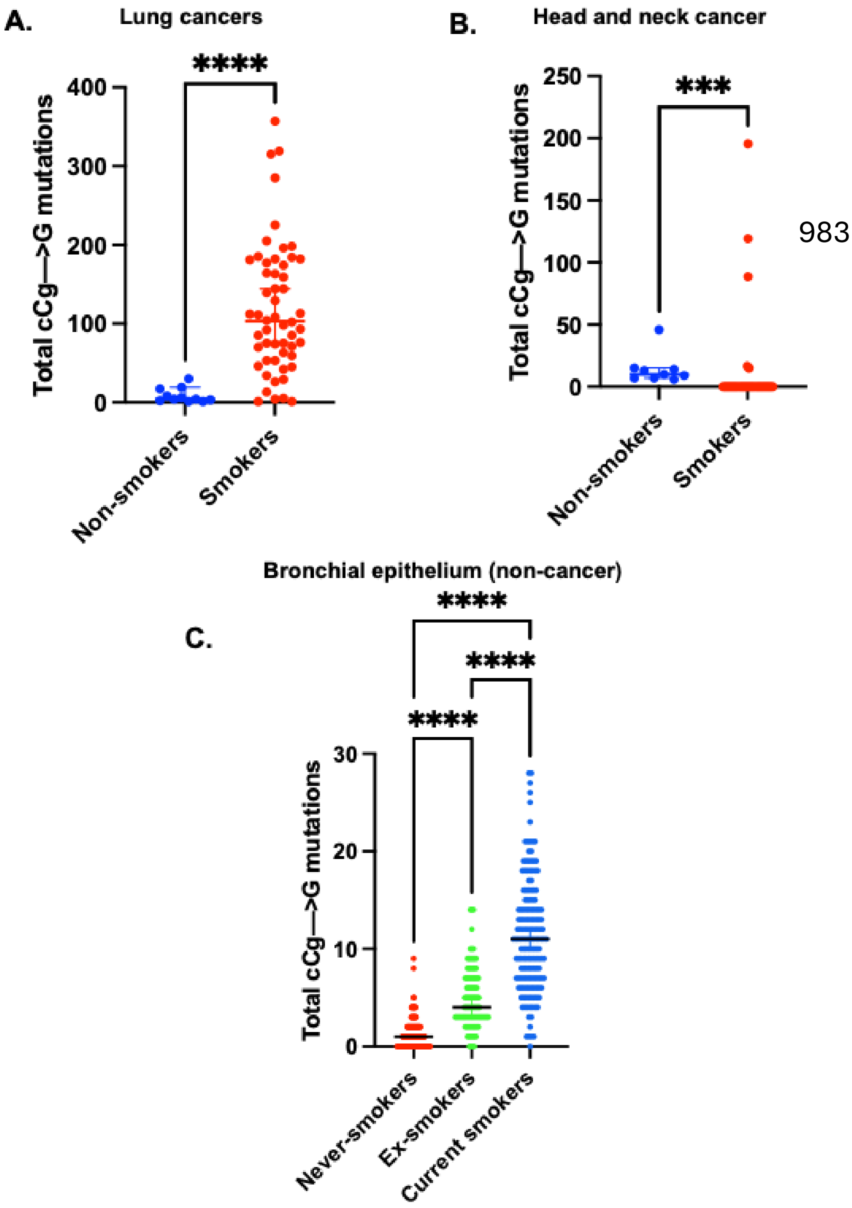

<u>Figure S4.</u> Correlation of cCg→G signature with smoking. A. cCg→G mutations in combined LUAD and LUSC datasets from PCAWG stratified according to smoking status. B. cCg→G mutations in HNSCC data from PCAWG stratified according to smoking status. For both A and B, smoking metadata was obtained from PCAWG. C. Analysis of cCg→G mutation loads in single-cell sequenced datasets from bronchial epithelia of current-, ex-, and never-smokers. Mutation calls and metadata was obtained from [69]. Asterisks represent p-value <0.05 based on an unpaired t-test.

bioRxiv preprint doi: https://doi.org/10.1101/2025.03.18.643935; this version posted March 18, 2025. The copyright holder has placed this preprint (which was not certified by peer review) in the Public Domain. It is no longer restricted by copyright. Anyone can legally share, reuse, remix, or adapt this material for any purpose without crediting the original authors.

Table S1: Strains and primers used in the study

Table S2: Source data for Figure 1C, 1E Figure S1.

Table S3: Total isolates analyzed via whole genome sequencing for the present study.

Table S4: Source data for Figure 2. MG induced-indels within ±10bp context

Table S5: Source data for Figure 3A.

Table S6: Source data for Figure 3B.

Table S7: Source data for Figure 5.

Table S8: Source data for Figure 6A.

Table S9: Source data for Figure 6B.

Table S10: Source data for Figure S4.
